# Supplementary material for: SOX9-dependent fibrosis drives renal function in nephronophthisis
Source: EMBO Mol Med. 2025 Apr 10;17(6):1238–58. doi: 10.1038/s44321-025-00233-3 (PMC12162883; doi:10.1038/s44321-025-00233-3)
Supplement: Supplementary file 1 — Appendix [file 44321_2025_233_MOESM1_ESM.pdf]

Appendix for “SOX9-dependent fibrosis drives renal function in nephronophthisis”

TABLE OF CONTENTS

1. **Table of contents……………………………………..Page1**
2. **Appendix Figure S1…………………………………..Page2**
3. **Appendix Figure S2…………………………………..Page3**
4. **Appendix Figure S3…………………………………..Page4**
5. **Appendix Figure S4…………………………………..Page5**
6. **Appendix Figure S5…………………………………..Page6**
7. **Appendix Figure S6…………………………………..Page7**
8. **Appendix Figure S7…………………………………..Page8**
9. **Appendix Figure S8…………………………………..Page9**
10. **Appendix Figure S9…………………………………..Page10**
11. **Appendix Figure S10…………………………………Page11**
12. **Appendix Figure S11…………………………………Page12**

Appendix Figure S1

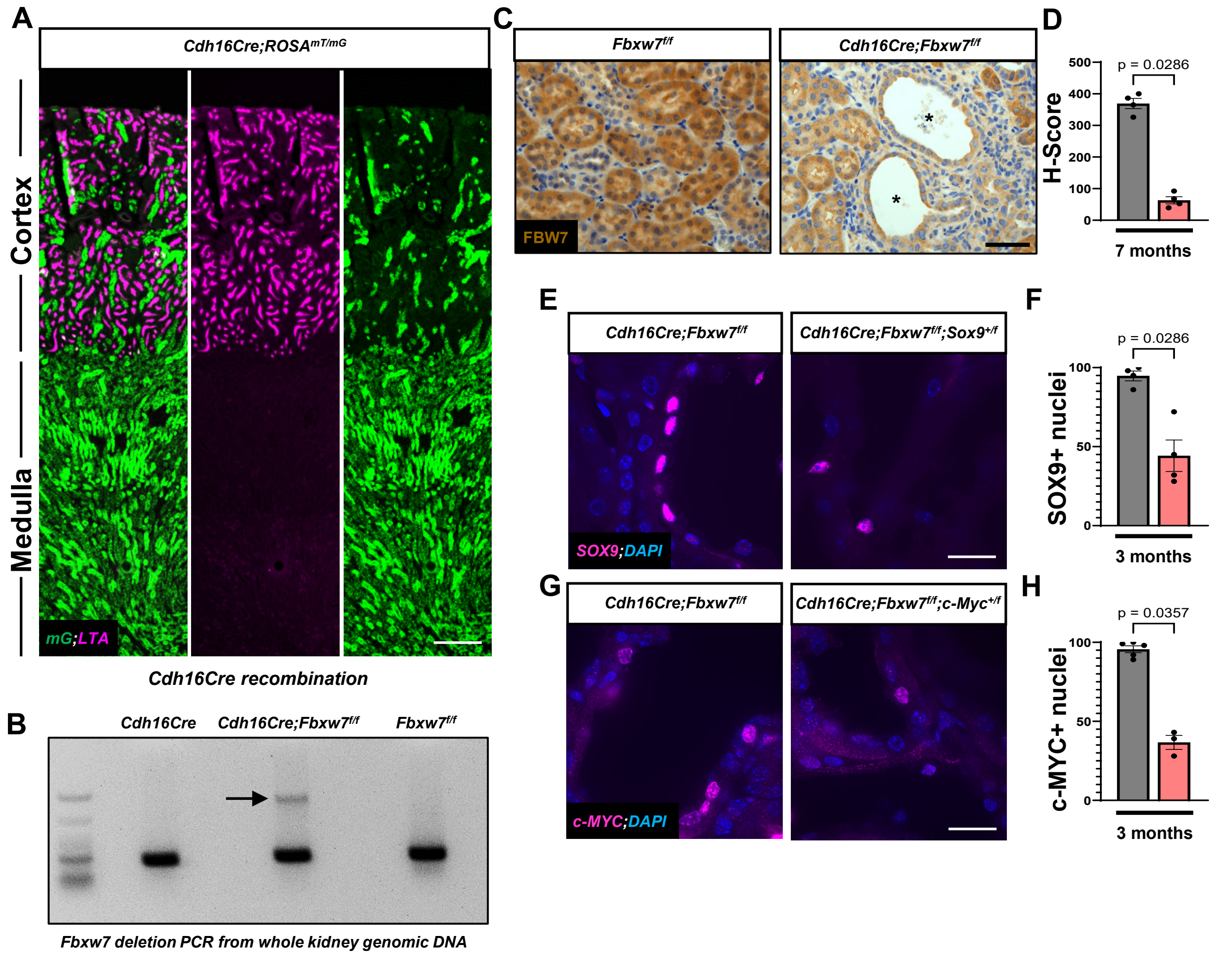

Appendix Figure S1: Characterization of *Cdh16Cre*-mediated *Fbxw7, Sox9, and c-Myc* deletion and recombination efficiency. (A) Representative images of *Cdh16Cre;ROSA$^{mT/mG}$* kidneys stained with LTA from P16 pups. *Cdh16Cre;ROSA$^{mT/mG}$* show predominant *Cre* activity in the medullar region and partial activity in the cortex region of the kidney (mG+, green). Scale bar: 100 µm. (B) PCR from DNA isolated from the whole kidney showed a 662 bp band, confirming *Cre*-mediated excision of exons 5 and 6 of *Fbxw7*. (C-D) FBW7 staining and quantification from 7-month-old *Fbxw7$^{f/f}$* and *Cdh16Cre;Fbxw7$^{f/f}$* kidneys. Scale bar: 200 µm. (D) Each data point represents the H-Score per animal from the cystic tubules (n=4). Statistical analysis was performed using the Mann-Whitney test and is presented as the mean ± SEM. (E-H) SOX9 and c-MYC staining and quantification from 3-month-old kidneys of *Cdh16Cre;Fbxw7$^{f/f}$* and *Cdh16Cre;Fbxw7$^{f/f}$;Sox9$^{+/f}$ or Cdh16Cre;Fbxw7$^{f/f}$;c-Myc$^{+/f}$* mice. Scale bar: 20 µm. (F and H) Each data point represents the percent reduction in SOX9+ or c-MYC+ cells scored from cystic tubules per animal (n≥3). Statistical analysis was performed using the Mann-Whitney test and is presented as the mean ± SEM.

Appendix Figure S2

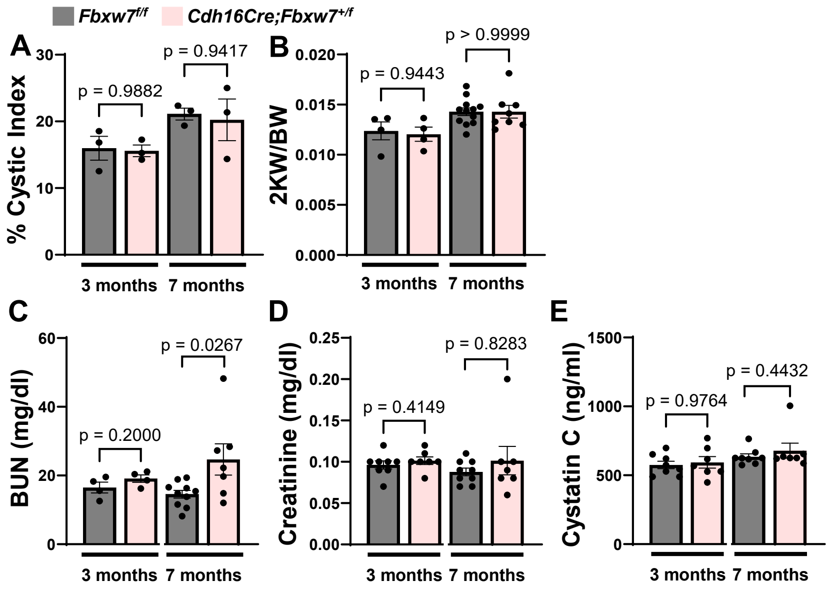

Appendix Figure S2: Heterozygous deletion of *Fbxw7* results in mild kidney function decline in aged mice. (A) Cystic index represented as the percentage of cystic area per kidney section (n=3), (B) 2KW/BW (n≥4), (C) Serum BUN (n≥4), (D) Creatinine (n≥7), and (E) Cytastin C (n≥7) from 3- and 7-month-old *Fbxw7$^{f/f}$* and *Cdh16Cre;Fbxw7$^{f/f}$* mice. Each data point represents one animal. Statistical analysis was performed using (A-B) one-way ANOVA followed by Šídák's multiple comparisons or (C-E) Mann-Whitney test and is presented as the mean ± SEM.

Appendix Figure S3

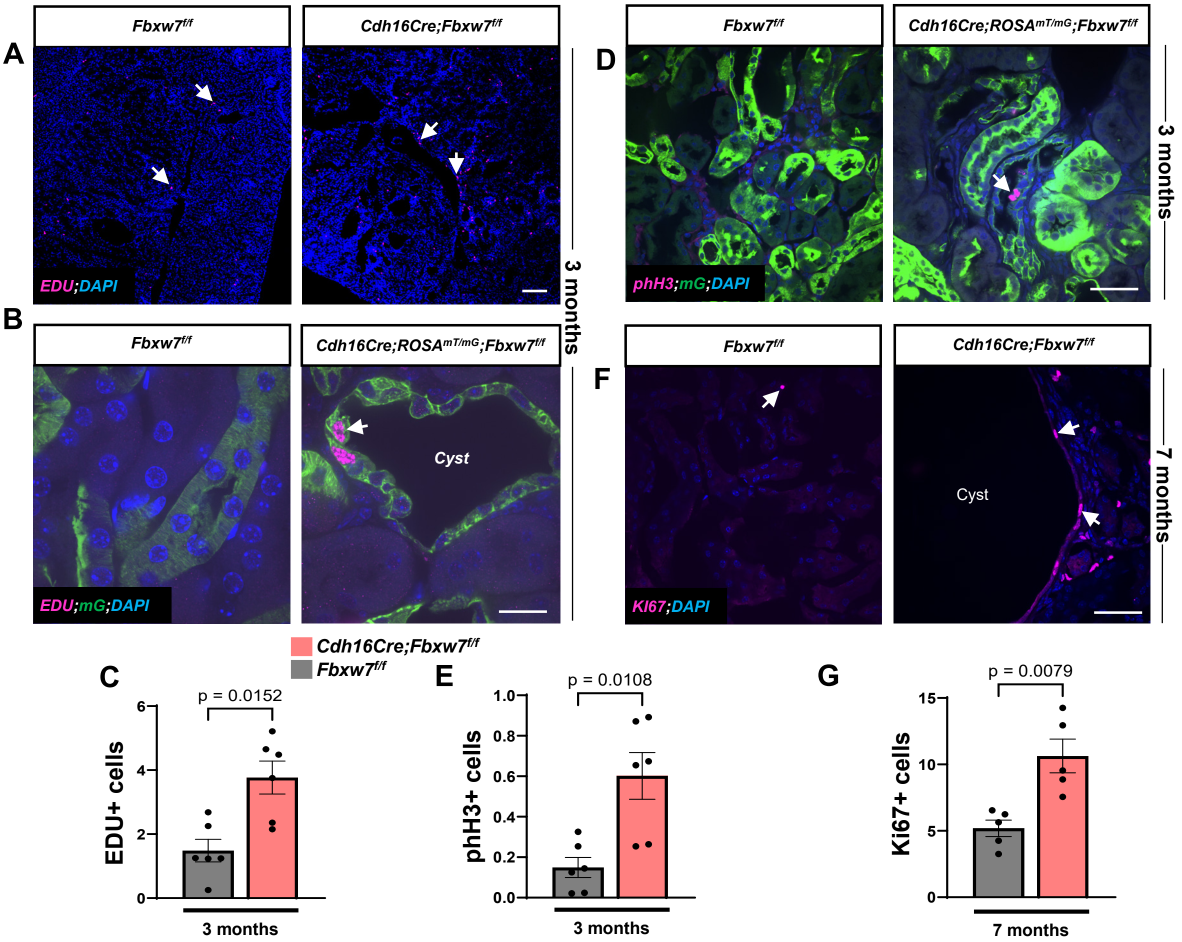

Appendix Figure S3: Deletion of *Fbxw7* increases proliferation in the kidney. (A-F) Representative images of (A) EdU+ cells (low magnification), (B) EdU+mG+ cells (high magnification), and (D) phospho-HISTONE H3 (pHH3)+mG+ cells from 3-month-old kidneys of *Fbxw7*$^{f/f}$ and *Cdh16Cre;Fbxw7*$^{f/f}$, with or without *ROSA*$^{mT/mG}$ background. White arrows show cells that are positive for respective proliferation markers. Nuclei are stained with DAPI (blue). Scale bar: (A) 100 µm, (B) 20 µm, and (D) 50 µm. (C and E) Each data point represents the average of (C) EdU+ or (E) pHH3+ tubular cells scored per animal (n=6). Statistical analysis was performed using the Mann-Whitney test and is presented as the mean ± SEM. (F-G) Representative images of Ki67+ cells from 7-month-old kidneys of *Fbxw7*$^{f/f}$ and *Cdh16Cre;Fbxw7*$^{f/f}$ mice. White arrows show Ki67+ (pink) cells of the cystic epithelium. Nuclei are stained with DAPI (blue). Scale bar: 50 µm. Each data point represents the average of Ki67+ cells scored per animal (n=5). Statistical analysis was performed using the Mann-Whitney test and is presented as the mean ± SEM.

Appendix Figure S4

A

Serum	Calcium	Phosphorus	Sodium	Potassium	Chloride	tCO2
	mg/dL	mg/dL	mmol/L	mmol/L	mmol/L	mmol/L
Fbxw7$^{f/f}$	11.06 ± 0.23	9.96 ± 0.43	160.00 ± 1.59	6.73 ± 0.22	112.86 ± 1.84	19.29 ± 1.29
Cdh16Cre;Fbxw7$^{f/f}$	11.51 ± 0.34	14.40 ± 1.35	159.29 ± 0.81	7.46 ± 0.25	111.86 ± 1.92	15.71 ± 0.84
p-value	0.2945	0.0083	0.6977	0.0488	0.7135	0.0384

Appendix Figure S4: Serum electrolytes. (A) Serum electrolytes from 7-month-old *Fbxw7$^{f/f}$* and *Cdh16Cre;Fbxw7$^{f/f}$* mice (n=7). Statistical analysis was performed using the Mann-Whitney test and is presented as the mean ± SEM. Analysis was done using serum on Abaxis vetscan.

Appendix Figure S5

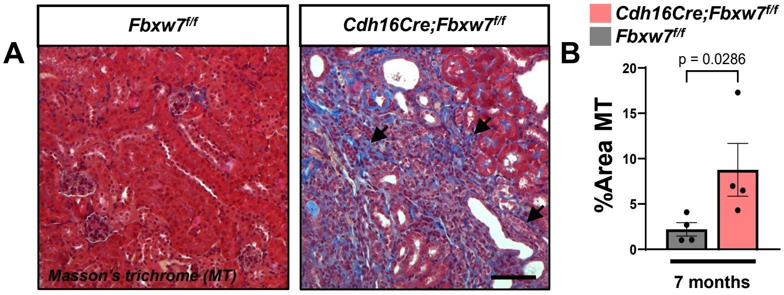

Appendix Figure S5: Loss of FBW7 results in excessive tubulointerstitial fibrosis in aged mice. (A-B) Representative images and quantification of (A-B) MT staining from 7-month-old kidneys of *Fbxw7*$^{f/f}$ and *Cdh16Cre;Fbxw7*$^{f/f}$ mice. (A) Black arrows show MT staining. Scale bar: (A) 200 μm. (B) Each data point represents the percent area of MT staining per kidney section per animal (n=4). Statistical analysis was performed using the Mann-Whitney test and is presented as the mean ± SEM.

Appendix Figure S6

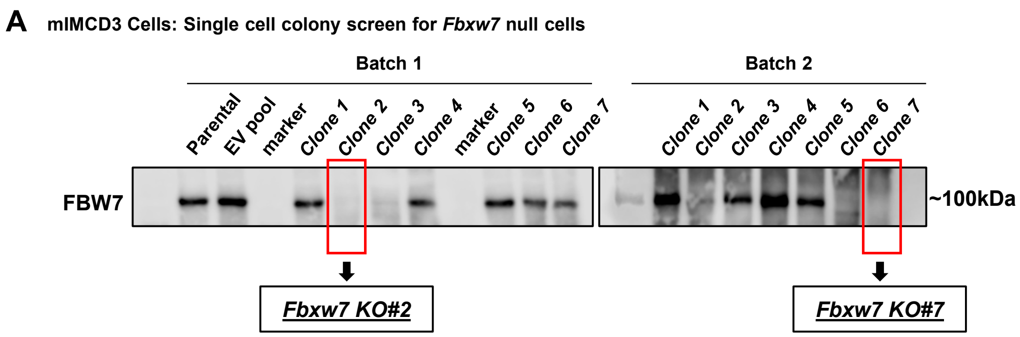

Appendix Figure S6: Characterization of *Fbxw7*-null mIMCD3 cell lines. (A) Immunoblots of FBW7 from different mIMCD3 clones generated via CRISPR-Cas9 gene editing using *Fbxw7*-specific sgRNA or controls. Equal protein concentrations were used to immunoprecipitate endogenous FBW7 using one FBW7 antibody, followed by detection on a Western blot using another FBW7 antibody. Clone 2 from batch 1 and Clone 7 from batch 2 were selected and labeled *Fbxw7 KO#2* and *Fbxw7 KO#7*, respectively.

Appendix Figure S7

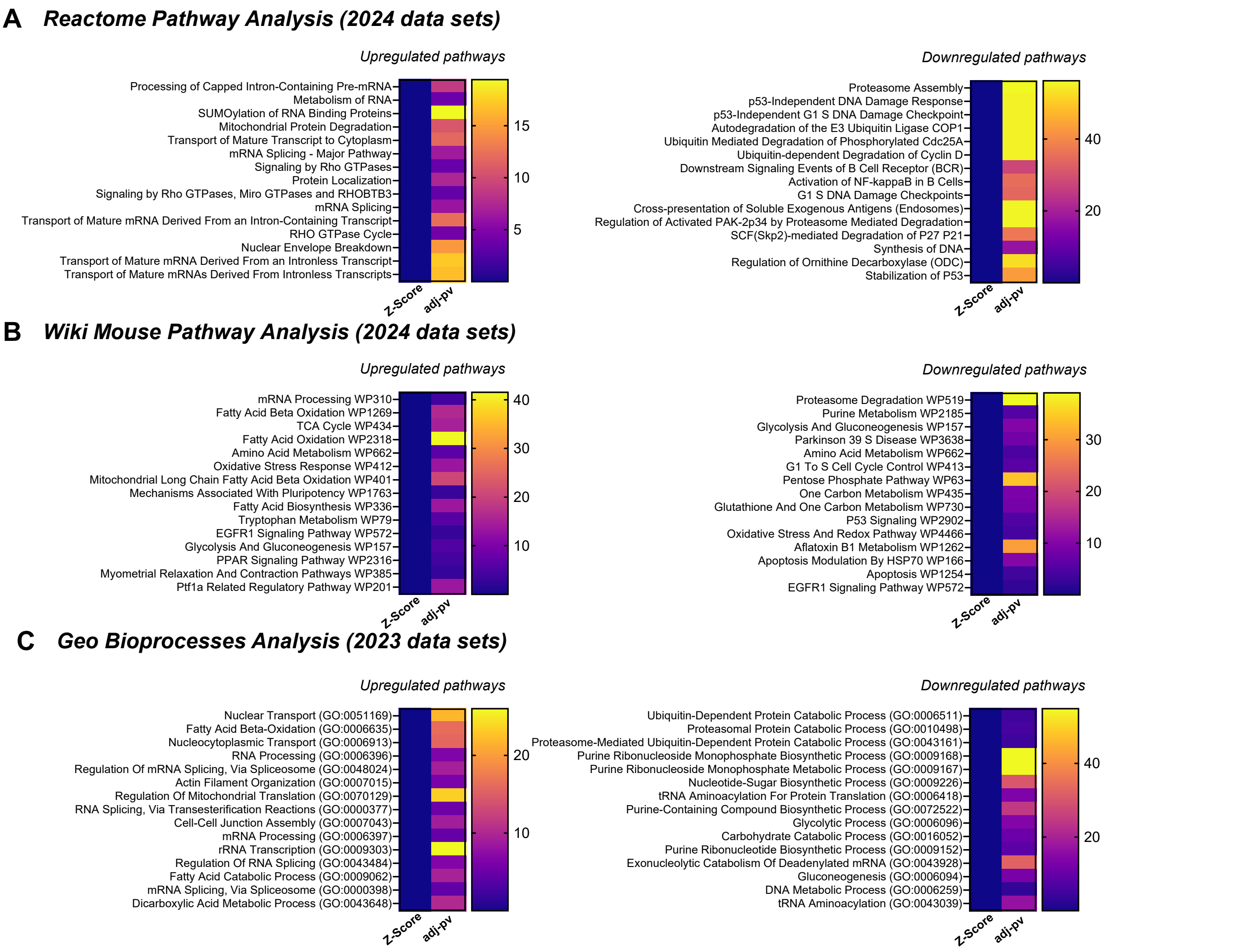

Appendix Figure S7: Pathway analysis of proteomic screen from *Fbxw7*-null mIMCD3 cell lines. All significantly upregulated or downregulated proteins in *Fbxw7*-null versus pooled empty-vector control mIMCD3 cell were used to do pathways analysis using Enrichr. The top 15 upregulated (left) or downregulated (right) pathways were represented using heat map (A) Reactome Pathway Analysis (2024 data sets), (B) Wiki Mouse Pathway Analysis (2024 data sets), and (C) Geo Bioprocesses Analysis (2023 data sets).

Appendix Figure S8

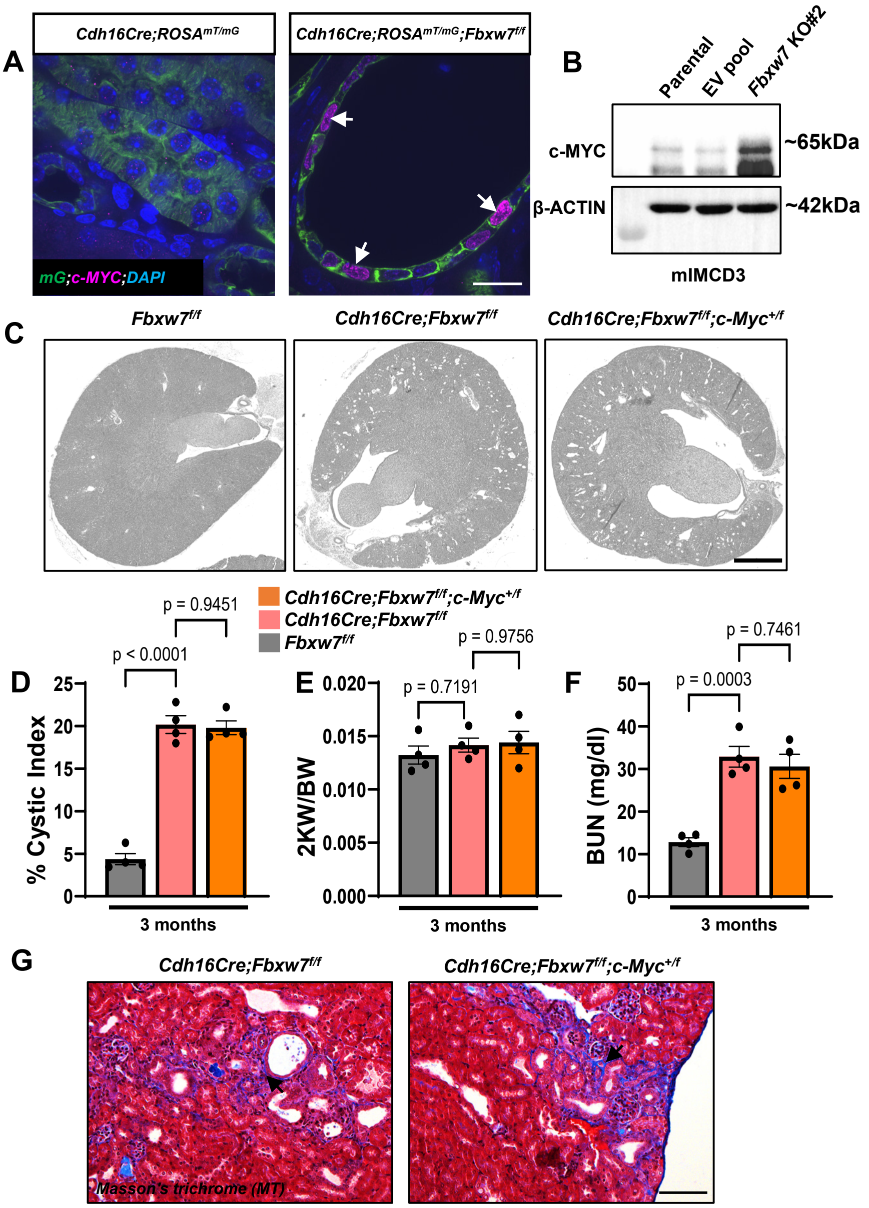

Appendix Figure S8: c-MYC does not contribute to early renal function decline in *Fbxw7*-deletion-based NPHP pathology. (A-B) c-MYC staining from 3-month-old kidneys of *Cdh16Cre;ROSA$^{mT/mG}$* and *Cdh16Cre;ROSA$^{mT/mG}$;Fbxw7$^{f/f}$* mice. (A) The white arrow shows nuclear c-Myc staining (pink) in mG+ tubules (green). Nuclei are stained with DAPI (blue). Scale bar: 50 µm. (B) Immunoblot of c-MYC from whole cell lysates of parental, EV pool, and *Fbxw7*-null (*Fbxw7 KO#2*) mIMCD3 cells. (C) Representative images of whole kidney section scan, (D) 2KW/BW (n=4), (E) cystic index represented as the percentage of cystic area per kidney section (n=4), and (F) Serum BUN (n=4) from 3-months-old *Fbxw7$^{f/f}$*, *Cdh16Cre;Fbxw7$^{f/f}$*, and *Cdh16Cre;Fbxw7$^{f/f}$;c-Myc$^{+/f}$* mice showing cyst progression and renal function. Each data point represents one animal. Statistical analysis was performed using one-way ANOVA followed by Šídák's multiple comparisons test and is presented as the mean ± SEM. Scale bar: 400 µm. (G) Representative images of MT from 3-month-old kidneys of *Cdh16Cre;Fbxw7$^{f/f}$*, and *Cdh16Cre;Fbxw7$^{f/f}$;c-Myc$^{+/f}$* mice. Black arrows show MT staining (purple). Scale bar: 200 µm.

Appendix Figure S9

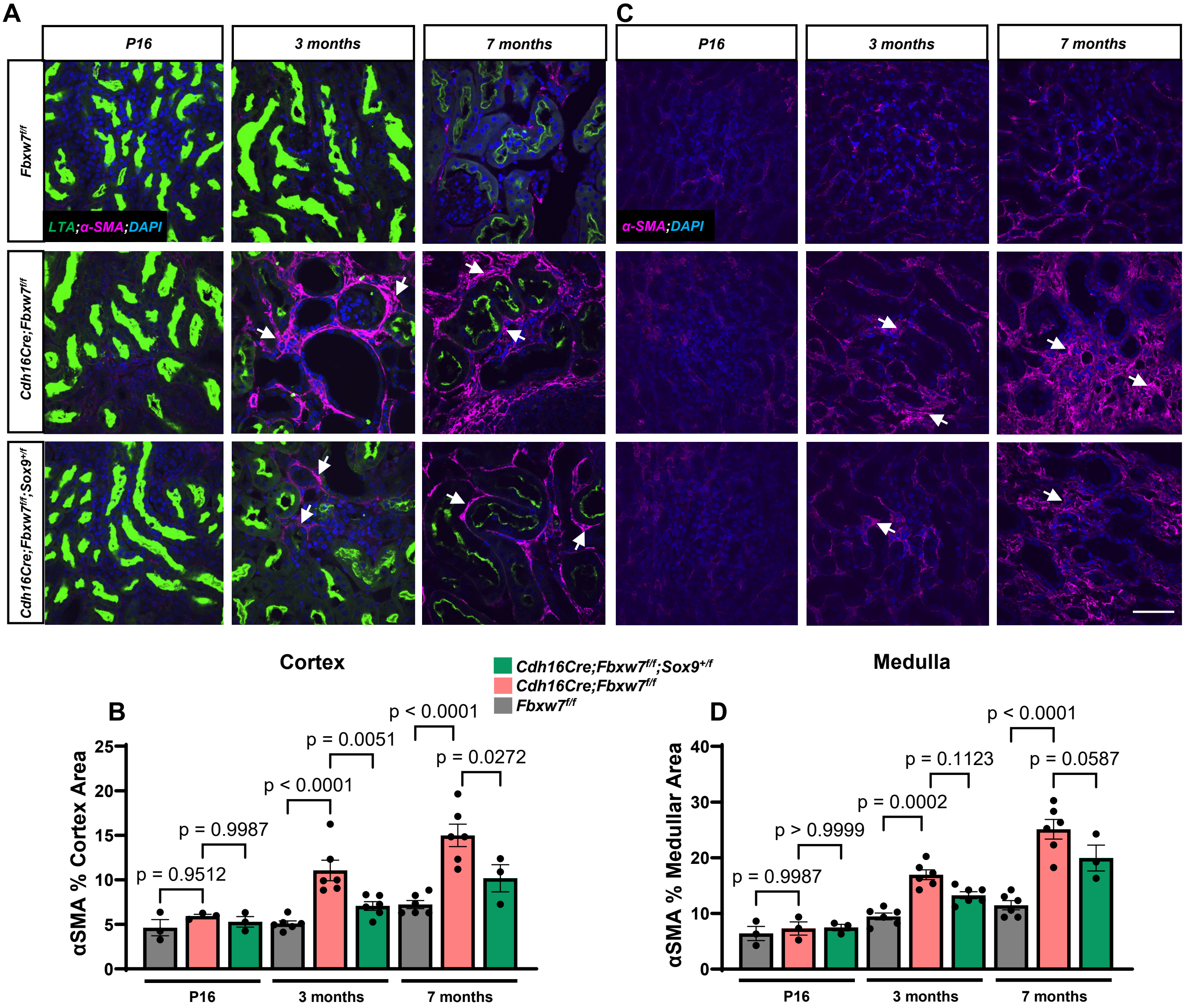

Appendix Figure S9: Spatio-temporal characterization of SOX9-dependent α-SMA expression in *Fbxw7*-deletion-based NPHP-like mouse model. (A-D) Representative images and quantification of α-SMA from different kidney regions of P16-, 3-, and 7-month-old *Fbxw7*$^{f/f}$ and *Cdh16Cre;Fbxw7*$^{f/f}$ mice. White arrows show α-SMA staining (pink). Nuclei are stained with DAPI (blue). Scale bar: 50 μm. (B and D) Each data point represents the α-SMA+ percent area from different kidney regions per animal (n≥3). Statistical analysis was performed using two-way ANOVA and is presented as the mean ± SEM.

Appendix Figure S10

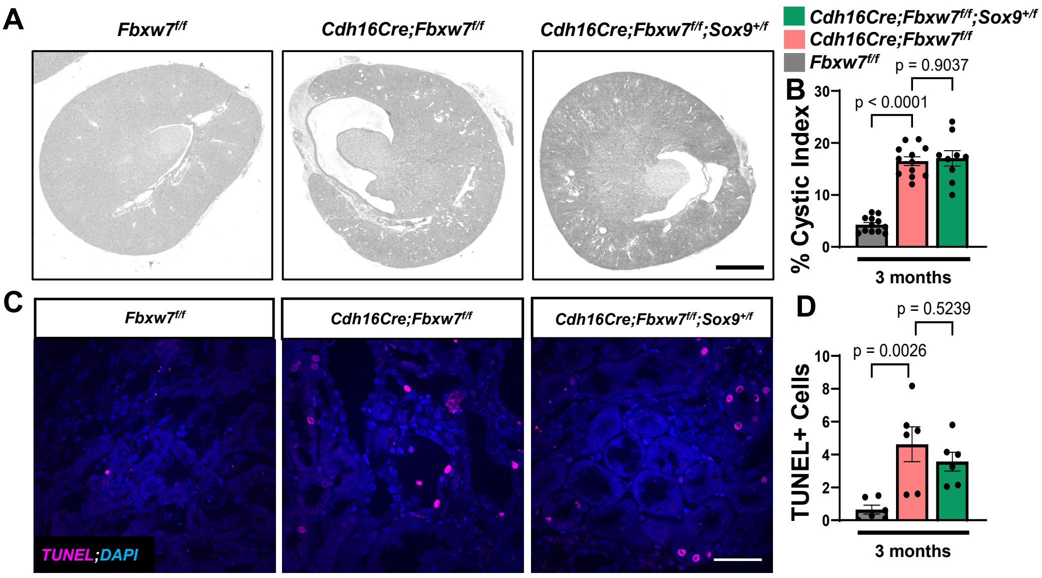

Appendix Figure S10: FBW7-SOX9 axis does not regulate cystic expansion and tubular degeneration. (A-B) Representative images and cystic index represented as the percentage of cystic area per kidney section from 3-month-old *Fbxw7$^{f/f}$*, *Cdh16Cre;Fbxw7$^{f/f}$*, and *Cdh16Cre;Fbxw7$^{f/f}$;Sox9$^{+/f}$* mice. Scale bar: 400 µm. (B) Each data point represents the percent kidney cystic index per animal (n≥9). Statistical analysis was performed using one-way ANOVA followed by Šídák's multiple comparisons test and is presented as the mean ± SEM. (C-D) Representative images of TUNEL staining and quantification from 3-month-old kidneys of *Fbxw7$^{f/f}$*, *Cdh16Cre;Fbxw7$^{f/f}$*, and *Cdh16Cre;Fbxw7$^{f/f}$;Sox9$^{+/f}$* mice. (C) TUNEL+ (pink) cells. Nuclei are stained with DAPI (blue). (D) Each data point represents the mean TUNEL+ cells scored per animal (n=7). Statistical analysis was performed using one-way ANOVA followed by Šídák's multiple comparisons test and is presented as the mean ± SEM.

Appendix Figure S11

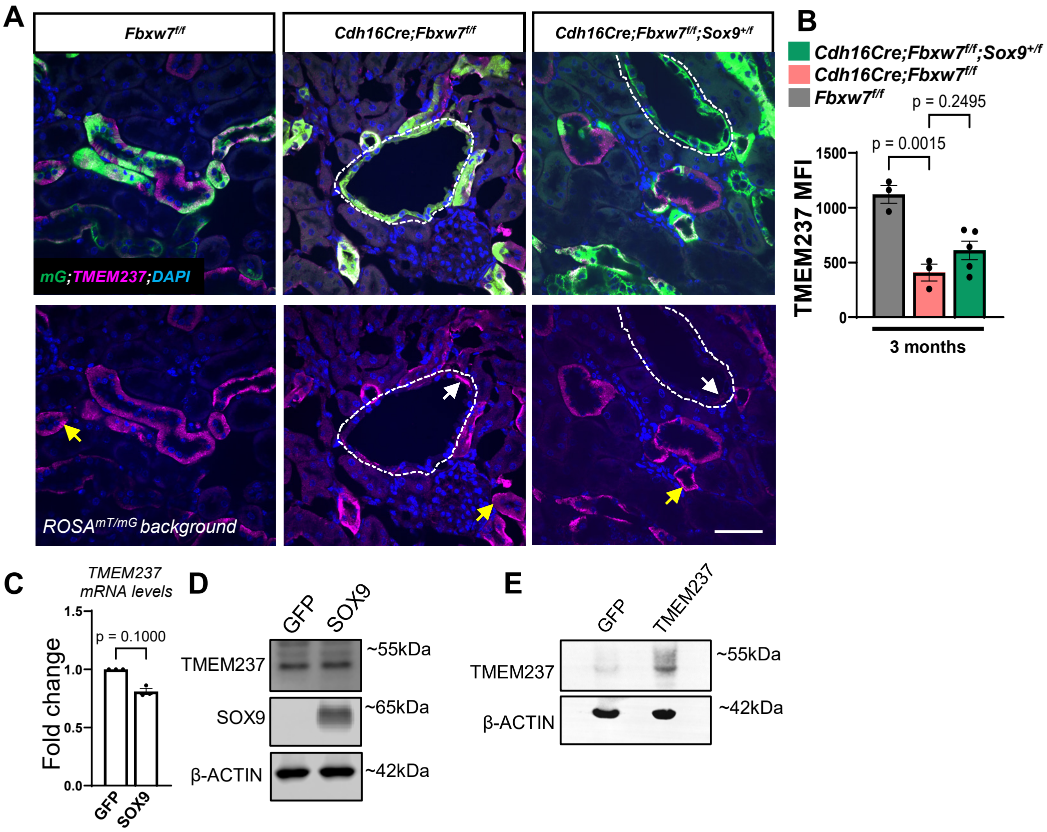

Appendix Figure S11: Downregulation of TMEM237 in cystic tubules upon loss of FBW7 is independent of SOX9 upregulation. (A-B) Representative images and quantification of TMEM237 from cystic tubules of 3-month-old *Fbxw7$^{f/f}$*, *Cdh16Cre;Fbxw7$^{f/f}$*, and *Cdh16Cre;Fbxw7$^{f/f}$;Sox9$^{+/f}$* mice with *ROSA$^{mT/mG}$* background. White arrows show the faint leftover signal of TMEM237 in cystic tubules, and yellow arrows show no changes in TMEM237 expression in non-cystic tubules. Scale bar: 50 µm. (B) Each data point represents an average of TMEM237 MFI in cystic tubules per animal (n≥3). Statistical analysis was performed using one-way ANOVA followed by Šídák's multiple comparisons test and is presented as the mean ± SEM. (C) mRNA levels and (D) Western blot for TMEM237 in HEK293T cells overexpressing transiently transfected SOX9 from n=3 experiments. (C) Statistical analysis was performed using the Mann-Whitney test and is presented as the mean ± SEM. (E) Western blot for TMEM237 from HEK293T cells overexpressing transiently transfected mouse TMEM237.
