# Supplementary material for: SOX9-dependent fibrosis drives renal function in nephronophthisis
Source: EMBO Mol Med. 2025 Apr 10;17(6):1238–58. doi: 10.1038/s44321-025-00233-3 (PMC12162883; doi:10.1038/s44321-025-00233-3)
Supplement: Supplementary file 2 — Table EV1 [file 44321_2025_233_MOESM2_ESM.docx]

**Table EV1: qPCR primers**

| *Gene name* | *Forward primer* | *Reverse primer* |
| --- | --- | --- |
| *Mouse Gapdh* | *AAAATGGTGAAGGTCGGTGTG* | *AATGAAGGGGTCGTTGATGG* |
| *Mouse Wnt4* | *AGACGTGCGAGAAACTCAAAG* | *GGAACTGGTATTGGCACTCCT* |
| *Human TMEM237* | *CCACCAGAGATGTGGCACTTAC* | *GGTTGGATAGCTGATCTCCTGC* |
| *Human β-ACTIN* | *CATCCTGCGTCTGGACCT* | *TAATGTCACGCACGATTTCC* |
