# Supplementary figures and images for: SOX9-dependent fibrosis drives renal function in nephronophthisis
Source: EMBO Mol Med. 2025 Apr 10;17(6):1238–58. doi: 10.1038/s44321-025-00233-3 (PMC12162883; doi:10.1038/s44321-025-00233-3)

❖ Figure 5B

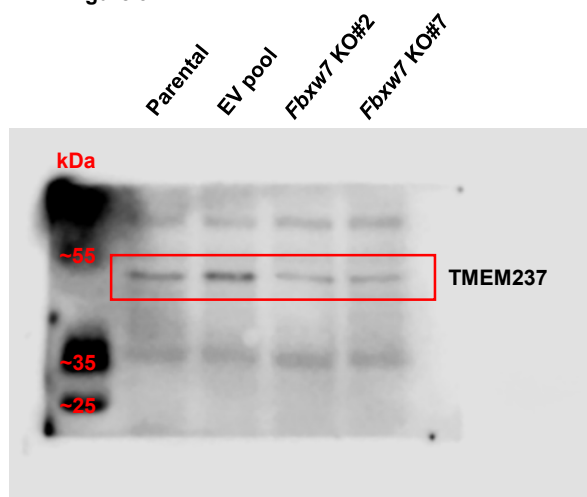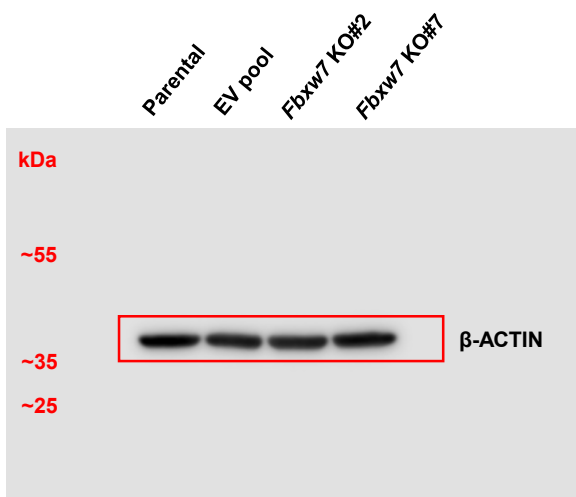

Supplement: Supplementary file 9 — Source data Fig. 5 [file 44321_2025_233_MOESM9_ESM.zip › Figure5 2/EMM-2024-20769-V3-Figure_5_Source_Data-sd 1.pdf]

❖ Figure 6C

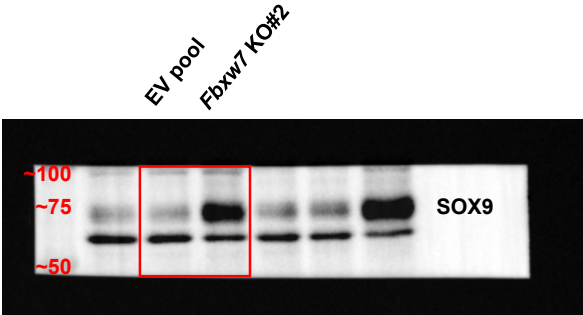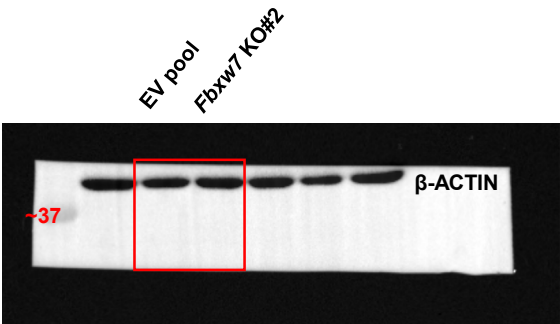

Supplement: Supplementary file 10 — Source data Fig. 6 [file 44321_2025_233_MOESM10_ESM.zip › Figure6 2/EMM-2024-20769-V3-Figure_6_Source_Data-sd 2.pdf]
