# Supplementary material for: SOX9-dependent fibrosis drives renal function in nephronophthisis
Source: EMBO Mol Med. 2025 Apr 10;17(6):1238–58. doi: 10.1038/s44321-025-00233-3 (PMC12162883; doi:10.1038/s44321-025-00233-3)

❖ **Figure 5B**

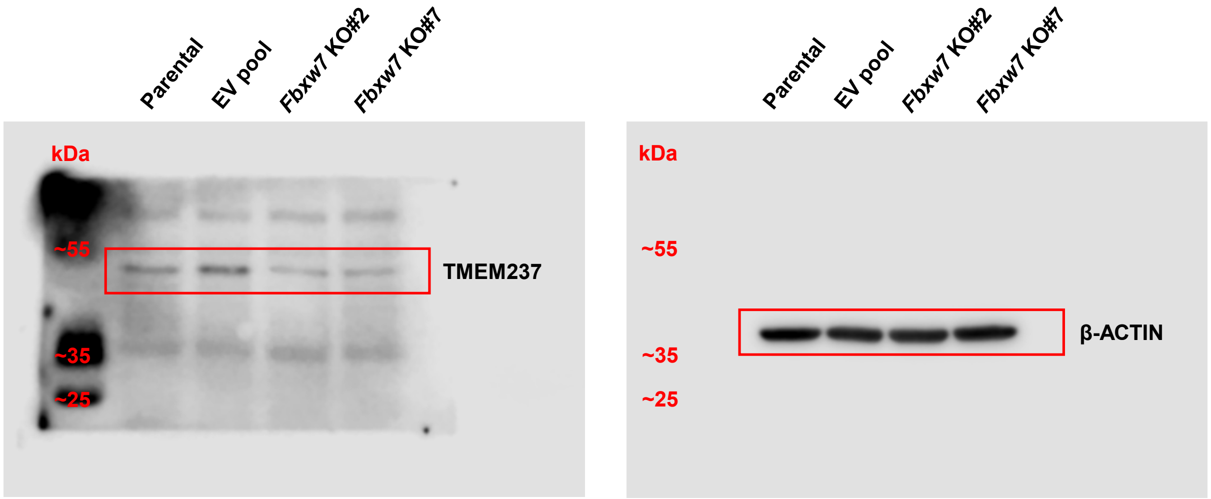
Parental
EV pool
Fbxw7 KO#2
Fbxw7 KO#7
kDa
~55
~35
~25
TMEM237
Parental
EV pool
Fbxw7 KO#2
Fbxw7 KO#7
kDa
~55
~35
~25
β-ACTIN

❖ **Figure 6C**

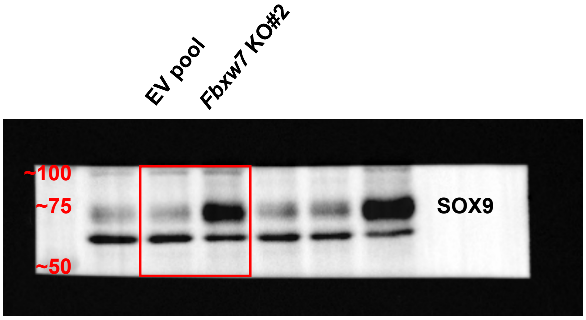

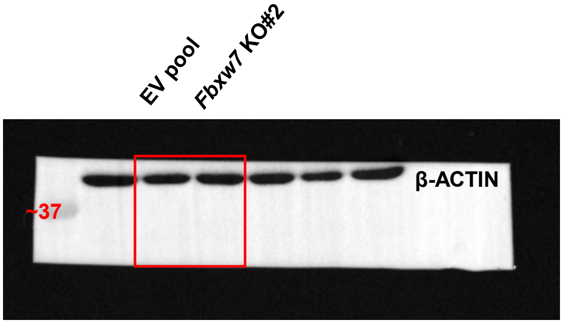

❖ **Appendix Figure S1B**

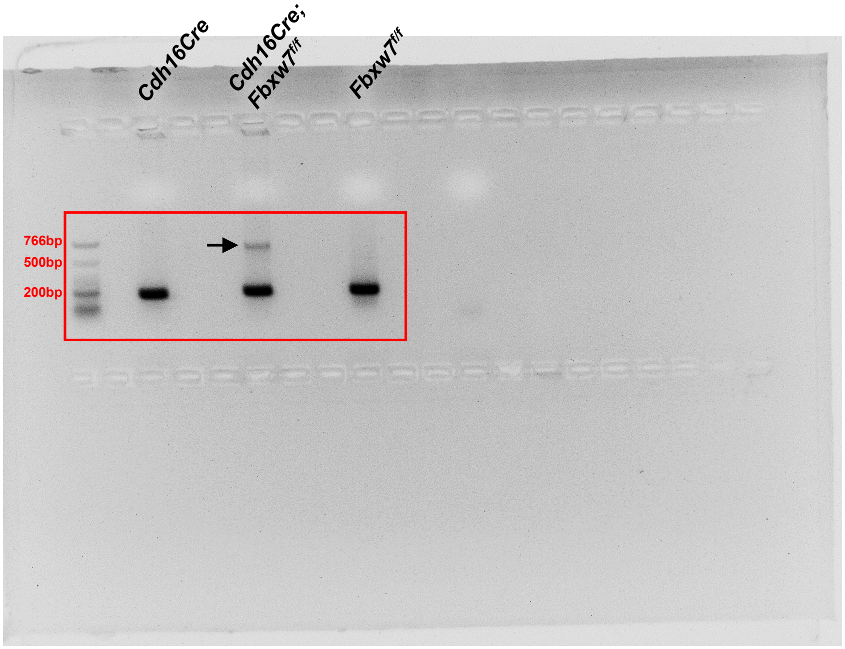

❖ **Appendix Figure S6**

Batch1

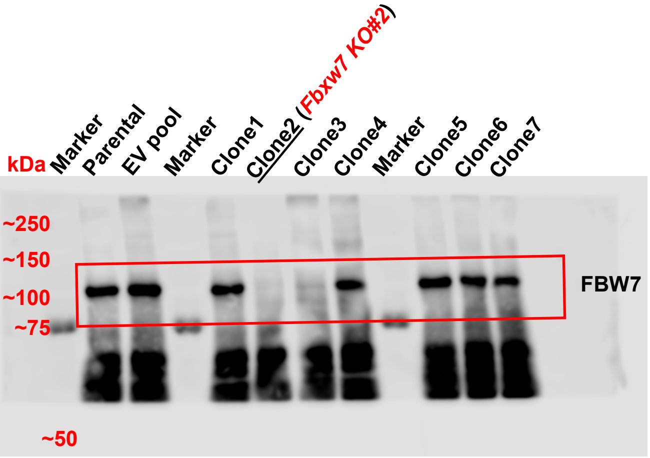

Batch2

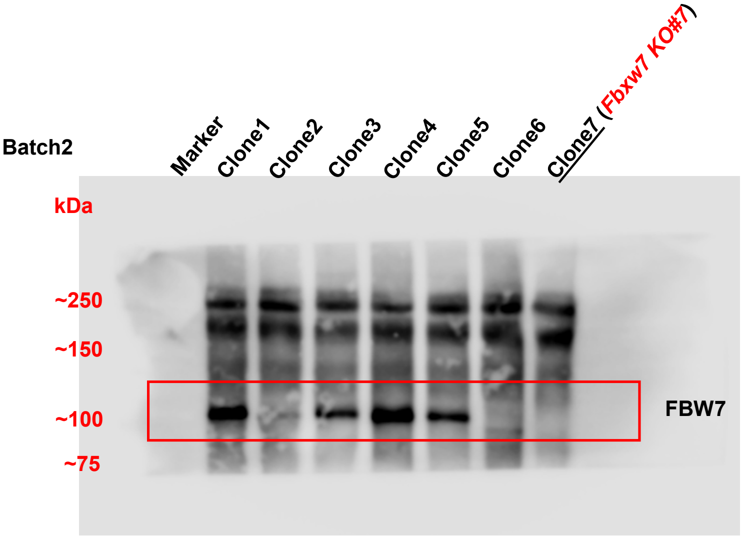

❖ Appendix Figure S8B

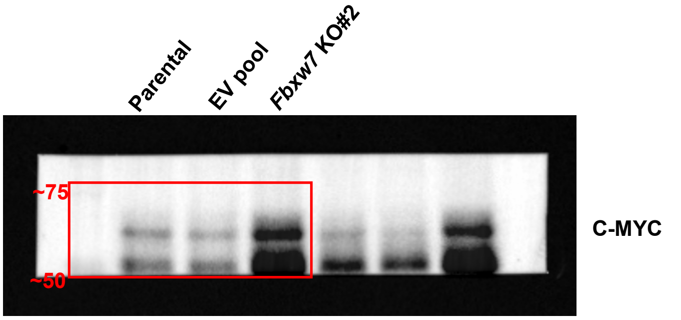

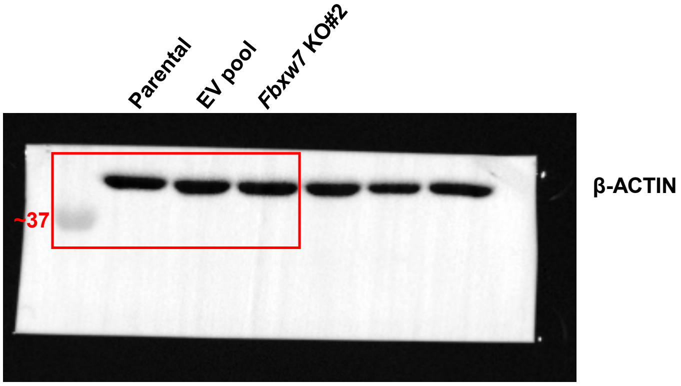

❖ **Appendix Figure S11D**

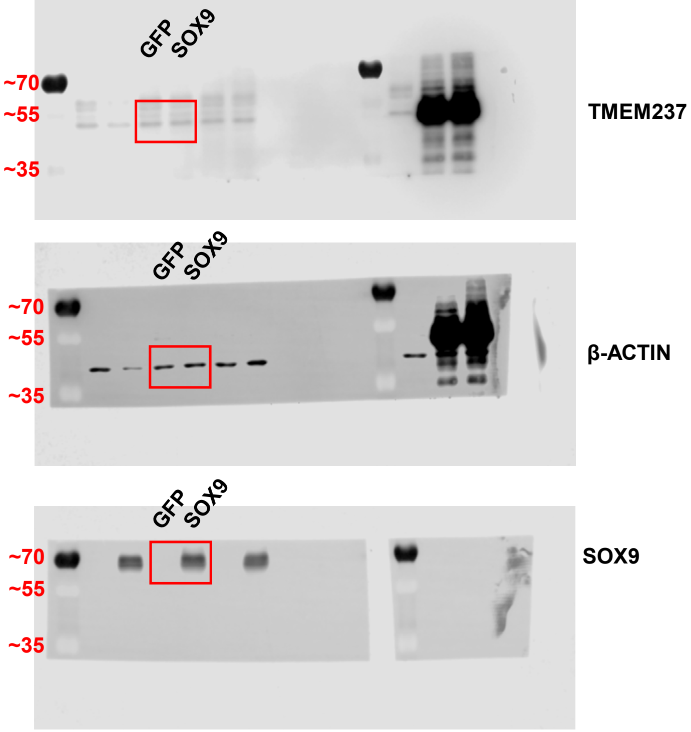

❖ **Appendix Figure S11E**

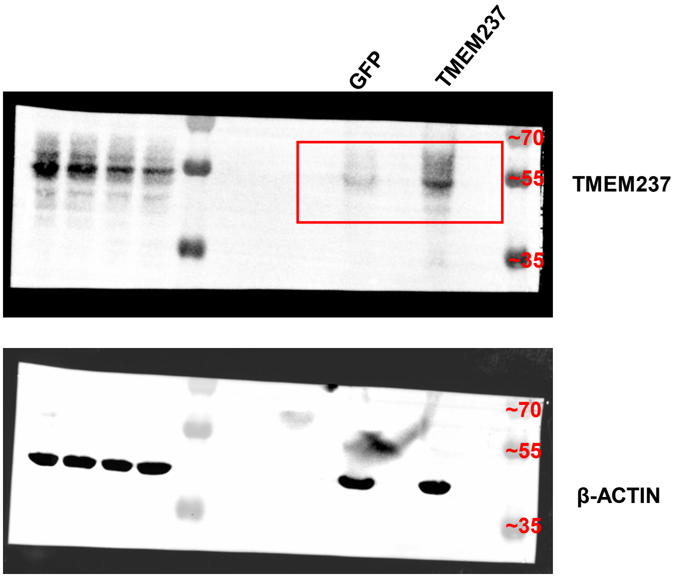

Supplement: Supplementary file 12 — Appendix Figure Source Data [file 44321_2025_233_MOESM12_ESM.zip › EMM-2024-20769-V3-Appendix_Figure_Source_Data-sd.pdf]
