# Supplementary material for: SOX9-dependent fibrosis drives renal function in nephronophthisis
Source: EMBO Mol Med. 2025 Apr 10;17(6):1238–58. doi: 10.1038/s44321-025-00233-3 (PMC12162883; doi:10.1038/s44321-025-00233-3)
Supplement: Supplementary file 13 — Expanded View Figures [file 44321_2025_233_MOESM13_ESM.pdf]

Expanded View Figures

Figure EV1. Deletion of *Fbxw7* results in cyst formation in different tubular segments of the kidney.

(**A**) Cystic index represented as the percentage of cystic area per kidney section from 1-, 3-, 7-, and 10-month-old *Fbxw7*$^{f/f}$ and *Cdh16Cre;Fbxw7*$^{f/f}$ mice. Each data point represents one animal ($n \geq 3$). Statistical analysis was performed using two-way ANOVA and is presented as the mean ± SEM. (**B**) Representative images of LTA+ and mG + cystic tubules in *Cdh16Cre;ROSA*$^{mT/mG}$*;Fbxw7*$^{f/f}$ (middle and right panels) and *Cdh16Cre;ROSA*$^{mT/mG}$ (left panel) mice. White arrows show cystic mG+ tubules that are LTA +. Nuclei are stained with DAPI (blue). Scale bar: 50 µm. (**C–J**) Representative images of immunofluorescence staining for (**C**, **D**) proximal tubule marker, Lotus tetragonolobus agglutinin (LTA), (**E**, **F**) thick ascending limb of the loop of Henle marker, NKCC2, and (**G**, **H**) collecting duct marker, Dolichos biflorus agglutinin (DBA) from 3- and 7-month-old kidneys of *Fbxw7*$^{f/f}$ and *Cdh16Cre;Fbxw7*$^{f/f}$ mice. White arrows show dilated or cystic tubular segments of the kidney that are positive for (**C**, **D**) LTA (green), (**E**, **F**) NKCC2 (pink), and (**G**, **H**) DBA (red). Nuclei are stained with DAPI (blue). Scale bar: 50 µm. (**I**, **J**) Pie chart displaying the percentage of cystic tubules distributed across different kidney segments from 3- and 7-month-old *Fbxw7*$^{f/f}$ and *Cdh16Cre;Fbxw7*$^{f/f}$ mice ($n \geq 3$).

 © The Author(s)

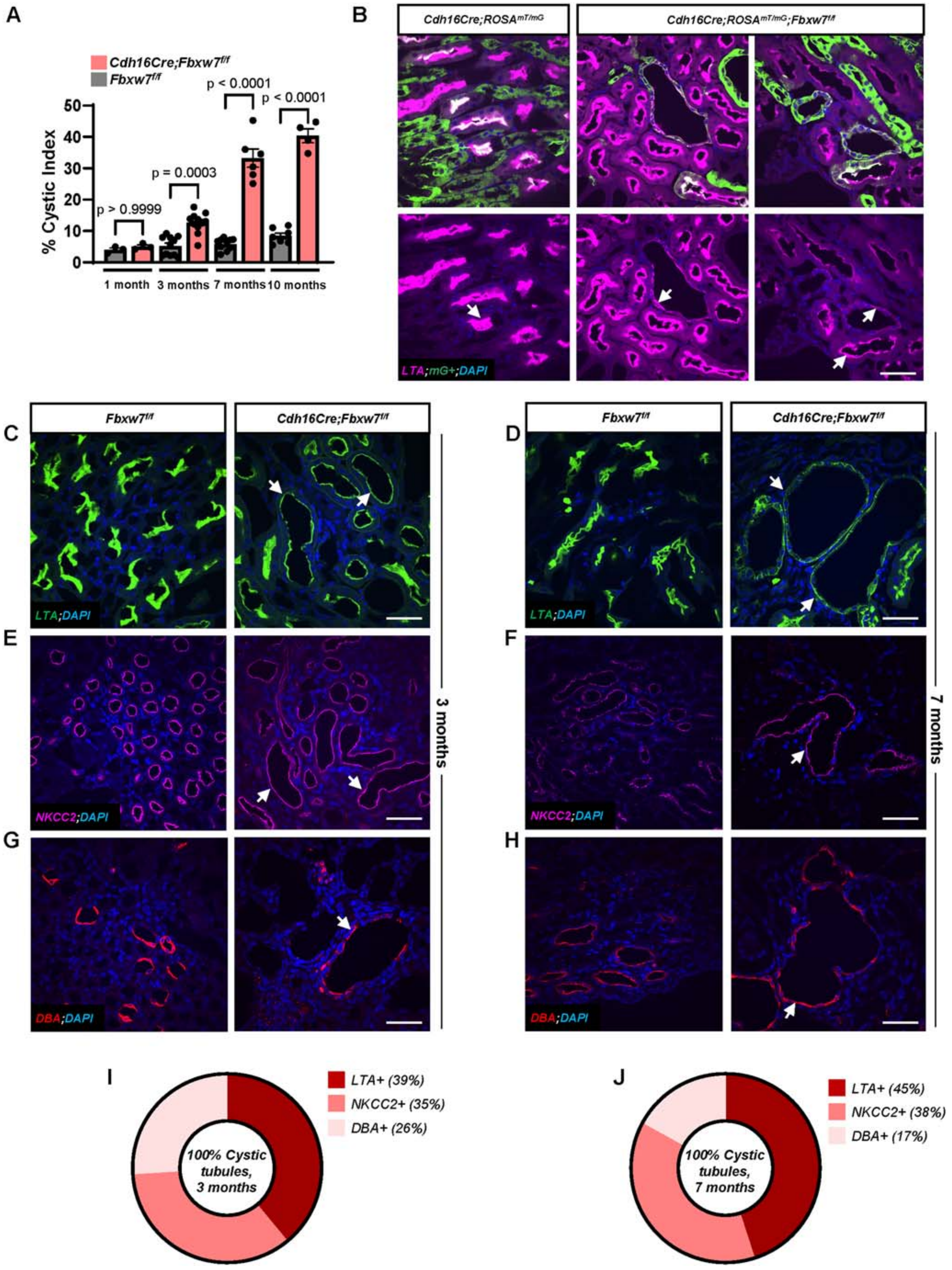
A
Cdh16Cre;Fbxw7f/f
Fbxw7f/f
% Cystic Index
p > 0.9999
p = 0.0003
p < 0.0001
p < 0.0001
1 month
3 months
7 months
10 months
B
Cdh16Cre;ROSAmT/mG
Cdh16Cre;ROSAmT/mG;Fbxw7f/f
LTA;mG+;DAPI
C
D
Fbxw7f/f
Cdh16Cre;Fbxw7f/f
LTA;DAPI
E
F
NKCC2;DAPI
G
H
DBA;DAPI
3 months
7 months
I
LTA+ (39%)
NKCC2+ (35%)
DBA+ (26%)
100% Cystic tubules, 3 months
J
LTA+ (45%)
NKCC2+ (38%)
DBA+ (17%)
100% Cystic tubules, 7 months

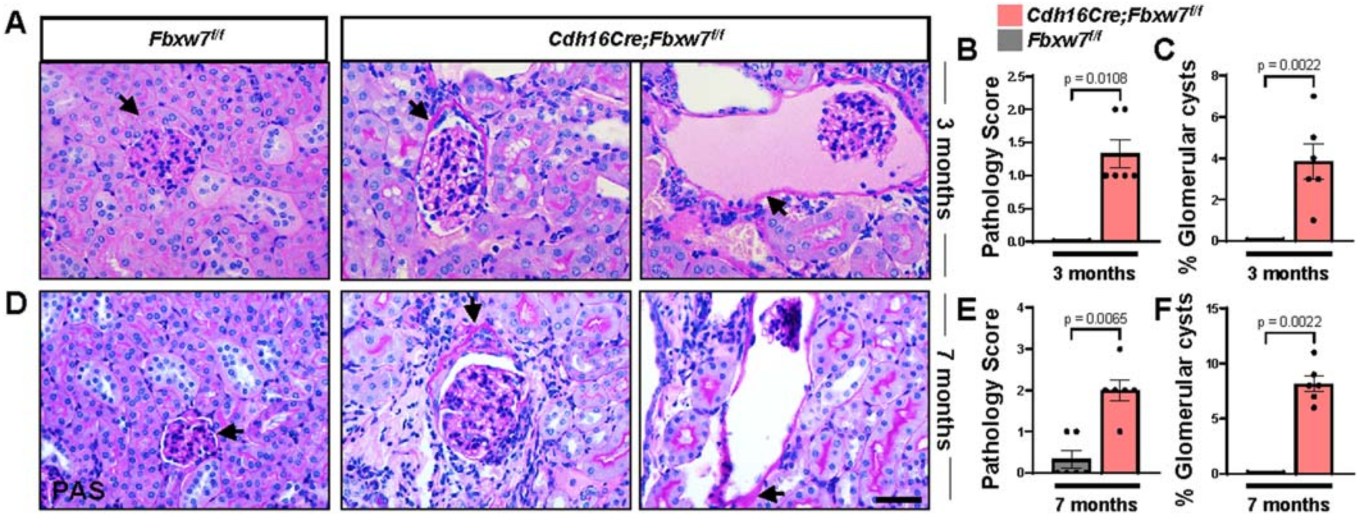

Figure EV2. Deletion of *Fbxw7* results in modest glomerular cysts and Bowman's capsule thickening.

(**A–F**) Representative images and quantification of glomerular cysts and mild thickening of Bowman's capsule (black arrows) using PAS staining from 3- and 7-month-old kidneys of *Fbxw7*$^{f/f}$ and *Cdh16Cre;Fbxw7*$^{f/f}$ mice. Nuclei are stained with Hematoxylin (blue). Scale bar: 200 µm. (**B**, **E**) Pathology score for the mild thickening of Bowman's capsule and (**C**, **F**) percentage of glomerular cysts scored per animal ($n = 6$). Statistical analysis was performed using the Mann–Whitney test and is presented as the mean ± SEM.

 © The Author(s)

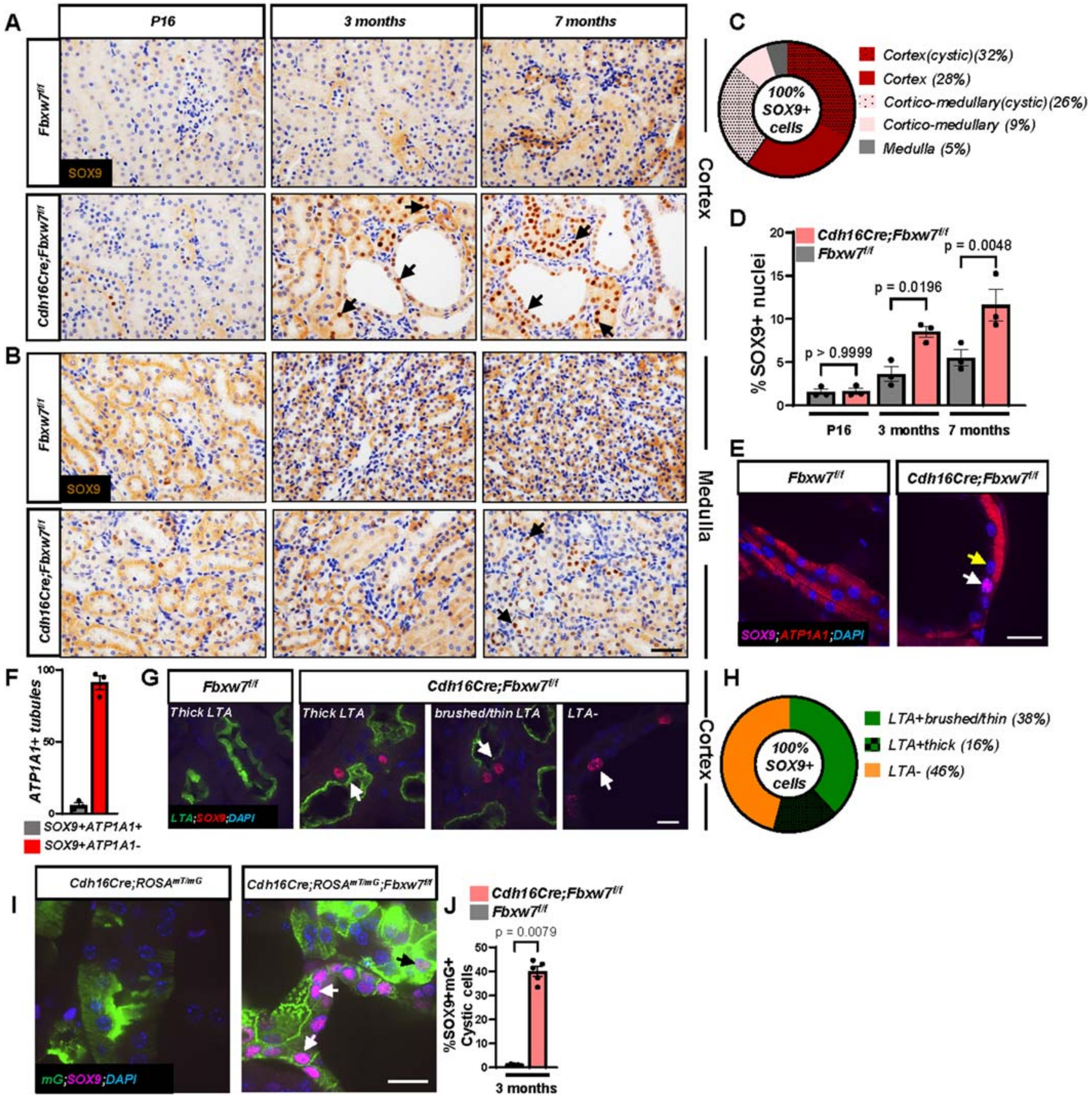

Figure EV3. Spatio-temporal characterization of increased SOX9 expression in *Fbxw7*-deletion-based NPHP-like mouse model.

(**A–D**) SOX9 staining and quantification from different kidney regions from P16-, 3-, and 7-month-old *Fbxw7*$^{f/f}$ and *Cdh16Cre;Fbxw7*$^{f/f}$ mice. (**A**, **B**) Black arrows show SOX9+ cells (brown). Nuclei are stained with Hematoxylin (blue). Scale bar: 100 µm. (**C**) Pie chart displaying the percentage SOX9+ cells distributed across different kidney regions from 3-month-old *Fbxw7*$^{f/f}$ and *Cdh16Cre;Fbxw7*$^{f/f}$ mice. (**D**) Each data point represents the percentage of SOX9+ cells scored per animal from P16-, 3-, and 7-month-old *Fbxw7*$^{f/f}$ and *Cdh16Cre;Fbxw7*$^{f/f}$ mice ($n = 3$). Statistical analysis was performed using two-way ANOVA and is presented as the mean ± SEM. (**E**, **F**) Representative images and quantification of SOX9+ cells within ATP1A1+ tubules that have lost ATP1A1 expression. (**E**) The white arrow shows the SOX + ATP1A1- cell, and the yellow arrow shows the SOX9-ATP1A1+ cell within the same cystic tubule. Nuclei are stained with DAPI (blue). Scale bar: 20 µm. (**F**) Each data point represents the percentage of SOX9+ cells scored per animal from ATP1A1+ tubules of 3-month-old *Cdh16Cre;Fbxw7*$^{f/f}$ mice. (**G**, **H**) Representative images and distribution of SOX9+ cells in LTA+normal-looking, LTA+brushed proximal tubules, and LTA- tubules. (**G**) The white arrow shows SOX9+ cells in respective tubules. Nuclei are stained with DAPI (blue). Scale bar: 20 µm. (**I**, **J**) SOX9 staining and quantification from 3-month-old kidneys of *Cdh16Cre;ROSA*$^{mT/mG}$ and *Cdh16Cre;ROSA*$^{mT/mG}$*;Fbxw7*$^{f/f}$ mice. (**I**) The white and black arrows show nuclear SOX9 staining (pink) in mG+ cystic and non-cystic tubules (green), respectively. Nuclei are stained with DAPI (blue). Scale bar: 20 µm. (**J**) Each data point represents the percentage of SOX9+ cells scored from mG+ cytic tubules per animal ($n = 5$). Statistical analysis was performed using the Mann–Whitney test and is presented as the mean ± SEM.
